# Amygdalin-Doped Biopolymer Composites as Potential Wound Dressing Films: In Vitro Study on *E. coli* and *S. aureus*

**DOI:** 10.3390/gels11080609

**Published:** 2025-08-02

**Authors:** Dorinel Okolišan, Gabriela Vlase, Mihaela Maria Budiul, Mariana Adina Matica, Titus Vlase

**Affiliations:** 1 Research Center for Thermal Analyzes in Environmental Problems, West University of Timişoara, 16 Johann Heinrich Pestalozzi Street, 300115 Timişoara, Romania; dorinel.okolisan11@e-uvt.ro (D.O.); gabriela.vlase@e-uvt.ro (G.V.); mihaela.budiul@e-uvt.ro (M.M.B.); 2 Institute for Advanced Environmental Research, West University of Timişoara (ICAM–WUT), 4 Oituz Street, 300086 Timişoara, Romania; 3 Department of Biology, Faculty of Chemistry, Biology, Geography, West University of Timișoara, 16 Johann Heinrich Pestalozzi Street, 300115 Timişoara, Romania; mariana.matica@e-uvt.ro

**Keywords:** amygdalin, polysaccharides, biopolymers, wound dressing, films

## Abstract

Biopolymer films doped with active substances may become a promising alternative to traditional dressings for skin wounds, as they can deliver drugs while maintaining wound moisture, thus contributing to the healing process. This article describes the preparation of amygdalin-doped biopolymer films for in vitro testing against the bacterial strains typical of chronic wounds: *E. coli* and *S. aureus*. Thus, FTIR characterization suggests minimal chemical interaction between amygdalin and the biopolymer matrix components, indicating potential compatibility, while thermogravimetric analysis highlights the thermal behavior of the films as well as the influence of the polymer matrix composition on the amount of bound water and the shift of *T_peak_* value for the decomposition process of the base polymer. Moreover, the identity of the secondary biopolymer (gelatin or CMC) significantly influences film morphology and antibacterial performance.

## 1. Introduction

As a complex tissue covering the entire external surface of the body, consisting of three distinct layers, dermis, epidermis, and hypodermis, the skin is the largest organ of the human body [1,2]. Acting as a barrier between the body and the external environment, the skin provides protection against UV rays, microorganisms, and physical damage, also being involved in the biogenesis of vitamin D and the process of thermoregulation of body temperature [3,4]. Any injury to the dermis layer that can disrupt the normal anatomy and function of skin can be defined as a skin wound [5], whether it is thermal or physical trauma, or as a result of a medical intervention [6]. From a clinical point of view, depending on the time required for healing, wounds can be classified as acute wounds, which heal gradually within a few days, and as chronic wounds, with a healing time of more than four weeks [7]. Prolonged healing of chronic wounds is generally due to inflammation, characterized by the inability of tissue to close and granulate, usually denoting a bacterial infection [8]. Bacterial colonization may be the first sign of wound infection; actual infection occurs only when colonization is coupled with additional elements, pathogenicity of certain bacterial species (such as *Staphylococcus aureus*), reduced vascular supply, and finally, the body’s immunological response [9]. Among the pathogens often found in chronic wounds, the following can be listed according to the literature: Gram-positive (*Staphylococcus aureus/MRSA* [10], *Streptococcus pyogenes* [11], *Enterococcus faecalis* [12], etc.) and Gram-negative (*Escherichia coli* [13], *Klebsiella pneumoniae* [14], etc.).

Wound healing occurs through an orderly process, consisting of four different phases: (1) the coagulation and hemostasis phase; (2) the inflammatory phase; (3) the proliferation period; and (4) the maturation phase [15], the promotion of which depends on the type of wound, the pathological conditions, but also on the nature of the dressing material [16]. Acting as a barrier that can promote the healing process, dressings are the ideal solution for protecting injured epidermal tissue [17] and are classified into four groups: conventional, biological, biosynthetic, and antimicrobial dressings. These should prevent skin drying and wound infection, thus contributing to wound healing [18]. Wound dressings can be designed as a drug delivery system, either in the form of thin films or in the form of gels [19].

Due to their biocompatibility and ability to naturally degrade, natural polymer-based dressings are widely used in wound care [20]. Biopolymer-based dressings can be effective solutions for wound care, due to their ability to form hydrogels that can absorb and release water, regulating excessive wound exudates and providing moisture, thus promoting healing [21]. This study focuses on three such biopolymers: κ-carrageenan, gelatin, and carboxymethyl cellulose (CMC). κ-carrageenan, a polysaccharide of marine origin, more precisely a sulfated galactan, is composed of alternating 1,3-linked β-d-galactopyranose-4-sulfate and 1,4-linked 3,6-anhydro-α-d-galactose units [22,23]. As a polymer of natural origin, obtained by hydrolytic degradation of collagen proteins [24], gelatin’s biodegradable properties make it suitable as a wound healing agent and drug carrier [25]. Although a polymer of synthetic origin, obtained in an alkaline medium by reacting cellulose with chloroacetate to substitute glucose units in the C2, C3 and C6 positions [26], carboxymethyl cellulose (CMC) has also found use in the field of wound dressing materials, in the form of hydrogels, films, fibers, etc. [27].

**Figure 1:**
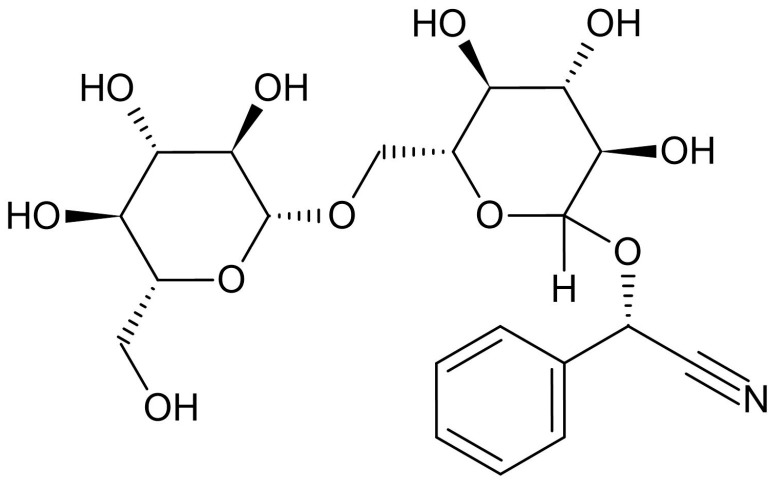
Chemical structure of amygdalin (adapted from [29]-CC BY 4.0).

Amygdalin, obtained by extraction from bitter almonds (*Prunus dulcis*) in the 1830s by Robiquet and Bourton-Chalard [28], is a cyanogenic glycoside present in olive, apple, sorghum, and grape seeds as well as in the kernels of apricots, peaches, and plums [29]. The chemical structure of amygdalin (Figure 1), or D-Mandelonitrile 6-O-β-d-glucosido-β-d-glucoside, is composed of two glucose units (interconnected by a glycosidic bond), a CN group and an benzene ring attached to a chiral C atom, thus being able to exist in either the R form (natural amygdalin) or the D form (neoamygdalin) [30]. This substance enjoyed great popularity among patients diagnosed with cancer in the 1970s, with a resurgence of interest in its consumption at the beginning of the 21st century [28]. Upon ingestion, amygdalin is hydrolyzed by intestinal β-glucosidase, thus releasing cyanide, which can lead to fatal cyanide poisoning, especially in large oral doses of amygdalin or when ingested concomitantly with fruit pits, almonds, or even high doses of vitamin C [31]. For several decades, amygdalin has enjoyed interest among scientists, whose efforts have succeeded in highlighting its therapeutic properties, including its antibacterial [32], immunomodulatory [33], and anti-inflammatory [34] properties, as well as its use as a treatment for various medical conditions such as asthma [35], colorectal cancer, etc. [36].

The need to develop new dermal wound dressings that are biodegradable, biocompatible, and at the same time able to enhance wound healing is the starting point of this study. Therefore, the aim was to obtain composite films based on κ-carrageenan, gelatin, and carboxymethyl cellulose with a potential utility in the field of wound dressings. Their characterization was carried out using FTIR and TG/DTG analysis. Due to its antibacterial properties [32], amygdalin was selected to be incorporated into these composite films, their bactericidal character being tested on *E. coli* and *S. aureus* cultures, these pathogens being often found in chronic wounds.

## 2. Results and Discussion

### 2.1. Film Synthesis

According to the synthesis methodology, two types of films were obtained, in which amygdalin was incorporated, in concentrations of 5, 10, 15, and 20 mg. This resulted in four type A films (A1, A2, A3, and A4), four type B films (B1, B2, B3, and B4), and two samples that did not contain active substance, the control samples, for each type of film, A0 and B0. As mentioned earlier, the drying of the films was carried out in an oven at 45 °C, the complete drying time being different in the case of the two types, 5 h in the case of type B and 24 h for type A. This difference in the time required for complete drying could be due to the composition of the polymer matrix itself; in this case, it seems that CMC facilitated their drying while gelatin caused an increase in this parameter. Also, the detachment of type B films from the bottom of the Petri dish was much easier; they practically almost “self-detached” during drying. Type A films are more sensitive to manipulation, even the tip of the tweezers with which they were manipulated during detachment being imprinted on their surface, as their appearance in Table 1 denotes. Regarding the physical appearance of the films, according to Table 1, it can be observed that they do not show any trace of crystallized active substance, so it can be said that the incorporation of the active substance was successfully achieved. Moreover, both type A and type B films, in addition to being transparent, present a smooth surface due to the reflection of light from their surface. Although both types of films are transparent, type B films present a much more pronounced degree of transparency compared to type A. Thus, it can be deduced that the nature of the secondary biopolymer has an impact on the “degree” of transparency of the synthesized films. Moreover, their elasticity may also be due to the secondary biopolymer; those with CMC being more elastic than those with gelatin (type A), which allows us to say that the presence of the active substance does not affect the integrity of the films. It should also be mentioned that those of type B also have a more pronounced adhesive character, which determined the creation of “wrinkles” when the films were peeled off, as can be seen in the case of films B0, B1, and B2.

### 2.2. FTIR Analysis

- *Base Components of Films*

**Figure 2:**
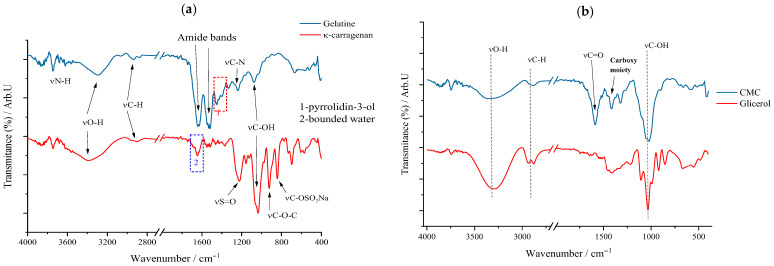
Graphical representation of FTIR spectra for κ-carrageenan and gelatin (**a**) and CMC and glycerol (**b**).

For a good understanding of the FTIR spectrum of the synthesized patches, it was considered that the FTIR spectrum of the components that form their biopolymer base and the active substance itself should first be “deciphered”. Therefore, in the following sections, the FTIR spectrum for each substance used will be discussed and interpreted. Thus, for the marine biopolymer, κ-carrageenan, the animal-derived one, gelatin, the cellulose derivative (CMC), and the plasticizer, glycerol (Gly), the corresponding FTIR spectra are represented in Figure 2.

For κ-carrageenan (κc), the following peaks can be highlighted: the one at 1035 cm^−1^ attributed to the stretching vibration of the C–OH bonds as a result of the presence of hydroxyl groups on the galactan chain and the one at 927 cm^−1^ associated with the vibration of the glycosidic bond (–C–O–C–) between the constituent units. Moreover, these in turn can serve as “witnesses” of the belonging of this biopolymer to the polysaccharide class. Also, the sulfated nature of this polysaccharide can be highlighted by the stretching vibration of the interatomic C–SO_3_Na bond at 839 cm^−1^. Going into more detail into the interpretation of the FTIR spectrum of this biopolymer, it can be said that both νO–H, with a band in the range of 3000–3600 cm^−1^, and νC–H, from 2900 cm^−1^, can be linked to the molecular structure of κ-carrageenan, these interatomic bonds being the result of the aliphatic skeleton of this biopolymer itself. As for the blue quadrant on the κc spectrum, it denotes that the biopolymer presents a quantity of trapped/bound water [37], indicating the hygroscopic nature of this polysaccharide of marine origin [38].

Gelatin’s biopolymeric nature, based on amino acids linked by peptide bonds, is confirmed primarily by two amide bands: amide I at 1637 cm^−1^ (νC=O stretching) and amide II at 1570 cm^−1^ (N–H bending in the peptide bond). The peptide bond’s intramolecular conjugation imparts partial single-bond character to the C=O bond, causing its vibrational peak to shift to a lower wavenumber, as observed in gelatin’s FTIR spectrum. Additionally, a peak near 1200 cm^−1^ corresponds to C–N stretching, reinforcing the amide nature. The 1360–1470 cm^−1^ region reflects νC–C skeletal vibrations of the pyrrolidin-3-ol moiety, while O–H and C–H stretching vibrations arise from the amino acid residues within the biopolymer.

The polysaccharide nature of carboxymethyl cellulose (CMC) is indicated by characteristic peaks, including the glycosidic bond at 930 cm^−1^ and broad C–OH vibrations between 3000 and 3500 cm^−1^, along with C–H stretching at 2900 cm^−1^ and deformation vibrations of O–H and C–H groups around 1300–1400 cm^−1^. The peak at 1620 cm^−1^ corresponds to the carbonyl group of the carboxylic fragment, which shifts to a lower wavenumber, while the intensity of the 1432 cm^−1^ peak confirms the presence of CMC as its sodium salt through carboxylate vibrations. For glycerol, signals arise from its three hydroxyl groups producing O–H bond vibrations, and the aliphatic backbone generates C–H stretching; additionally, the C–OH stretching peak at 1050 cm^−1^ and δO–H and δC–H deformation bands between 1300 and 1400 cm^−1^ resemble those observed in cellulose derivatives.

**Figure 3:**
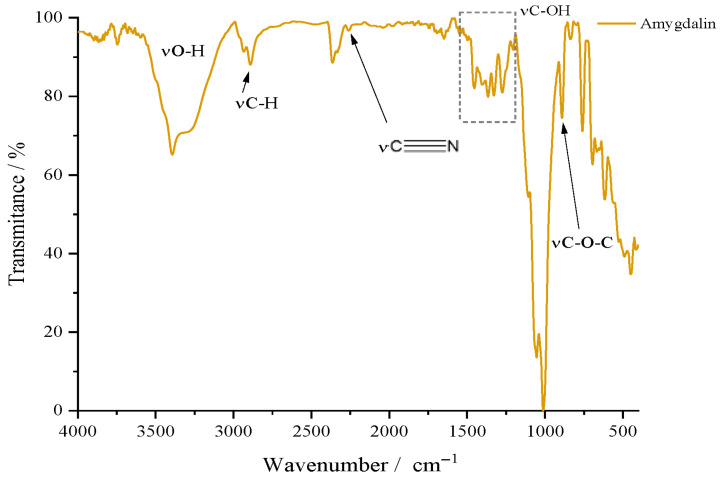
FTIR spectrum of amygdalin.

For the active substance, amygdalin (Amg), the results of the FTIR analysis are graphically represented in Figure 3. For amygdalin, the two glucopyranosyl units can be confirmed by the peak at 1000 cm^−1^ due to the stretching vibrations of the C–OH bond; the peak at 890 cm^−1^ for the glycosidic bond; the band in the 3000–3500 cm^−1^ range for νO–H; and last but not least, νC–H at 2900 cm^−1^. As for the benzene ring, it can be highlighted by the bands located in the gray quadrant, the 1300–1500 cm^−1^ area, due to the stretching vibrations of the C=C but also =C–H bonds. Below 1000 cm^−1^, two bands can be observed that can confirm the substitution mode of the benzene ring; thus, the intense one at 758 cm^−1^ and the medium intensity one at 450 cm^−1^ indicate the monosubstituted nature of benzene found in the molecular structure of amygdalin. As a compound containing the nitrile portion, it can be recognized by the appearance of a peak around 2100–2200 cm^−1^. The FTIR spectrum of amygdalin does not exhibit a strong nitrile peak around 2100–2200 cm^−1^ as expected. Instead, a medium-intensity peak is observed near 1250 cm^−1^, suggesting that the σ bond (C–N) is more detectable, while the π bonds may be overlapping with vibrations from the benzene skeleton, specifically the C=C and =C–H vibrations (gray quadrant).

Regarding the films in which amygdalin was incorporated, the results of the FTIR analysis will be discussed in the following paragraphs. Also, the control films will be compared with each other, and the films containing the active substance will be compared with the FTIR spectrum of amygdalin, but also with the corresponding control film. Given the large number of samples obtained, this approach to communicating/presenting the results was considered a simple and efficient approach, thus avoiding the communication of repetitive information.

- *Control Films (A0 and B0)*

**Figure 4:**
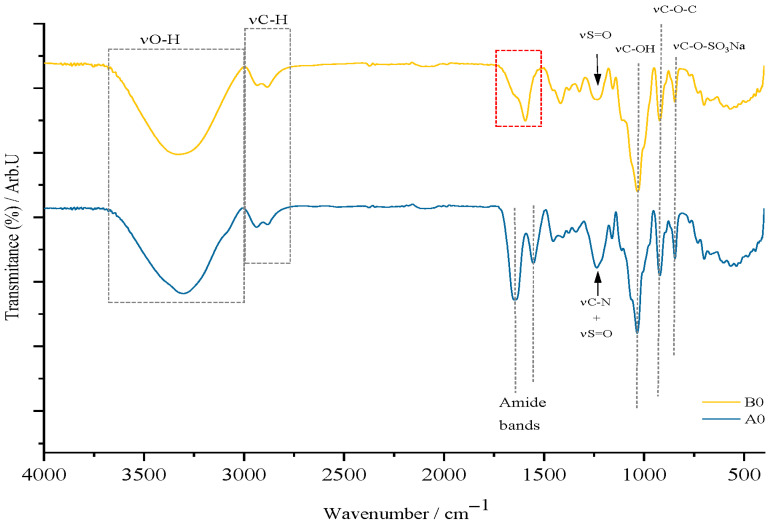
Graphical representation of the spectra for the control films, A0 and B0.

Figure 4 shows that films containing κ-carrageenan exhibit characteristic signals of this marine biopolymer, notably the C–O–SO_3_Na stretching vibration at 840 cm^−1^, confirming its unaltered presence in the films. In the control film (type A), gelatin is identified by amide bands linked to C=O stretching and N–H deformation in peptide bonds. The 1200 cm^−1^ signal corresponds to both νS=O and νC–N in type A, but only to νS=O in type B films. Type B films also display a medium-intensity signal around 1624 cm^−1^ (the red quadrant) attributed to bound water and the carboxyl group in carboxymethyl cellulose (CMC), with this signal shift retained. Both film types share common features such as O–H and C–H deformation bands (1300–1400 cm^−1^), glycosidic bond vibrations at 920 cm^−1^, C–OH vibrations at 1050 cm^−1^, and stretching vibrations for O–H and C–H.

- *Films Incorporating Active Substance (Types A and B)*

**Figure 5:**
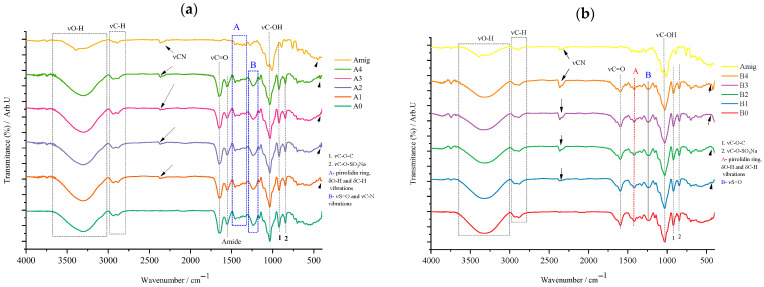
Comparative representation of FTIR spectra for type A (**a**) and type B (**b**) films with control films A0 and B0 and amygdalin.

For type A films (Figure 5a), one can observe the appearance of vibrations characteristic of both gelatin (designated in the blue quadrant A) and those characteristic of carrageenan, below 1000 cm^−1^. Thus, quadrant A denotes the skeletal vibration, more precisely νC–C of the pyrrolidin-3-ol portion of gelatin. Upon closer inspection of the spectra of the analyzed samples, one can see that this quadrant A is also extended over the FTIR spectrum of amygdalin, since in this area of the spectrum, the stretching vibration of the C=C and =C–H bonds in the benzene ring of amygdalin also occurs. The highlighting of the peak at 1244 cm^−1^ (blue quadrant B) was considered an important step because at this number two characteristic vibrations are found superimposed, namely the C–N stretching vibration of the peptide bonds in gelatin and, respectively, νS=O of κ-carrageenan. It can be said that the absence of this peak but also those characteristic of carrageenan, νC–O–C and νC–OSO_3_Na, could indicate possible interactions either between biopolymers or between the incorporated active substance and them, which is not the case of the analyzed samples. Both gelatin, glycerol, and κ-carrageenan contribute to the intensity of the peaks for νO–H, at cm^−1^, and νC–H at cm^−1^, respectively. In the case of type B films whose biopolymer matrix is formed by CMC and κ-carrageenan, the FTIR spectrum (Figure 5b) shows a characteristic peak that can be attributed with certainty only to the cellulose derivative, namely the one at 1432 cm^−1^ designated as A and which is due to the vibration of the carboxy fragment, COO^−^. This again reconfirms the nature of this derivative, being observed in all type B samples, and it can be a control indicating the unaltered presence of this biopolymer in all synthesized films. In the case of this type of film, the peak at 1244 cm^−1^ comes only from the S=O stretching vibration of κ-carrageenan. Again, as in the case of type A films, the appearance of characteristic κ-carrageenan peaks can be observed in the fingerprint area. It should be noted that the intensity of the νC–O–C peak, at 927 cm^−1^, is also contributed by the vibrations of the glycosidic bonds in CMC, the “visibility” of this peak indicating that the presence of amygdalin does not induce chemical modifications of these two biopolymers. Finally, around 2800–3500 cm^−1^, the characteristic peaks for the stretching vibrations of the O–H and C–H bonds can be found due to the presence of these interatomic bonds in the components of the polymeric matrix of type B films.

Regarding the active substance incorporated into the matrix of both types of films, the characteristic signals for the substitution mode of the benzene ring are visible for both type A and type B films. Furthermore, it can be observed that the peak attributed to the vibration of the glycosidic bond in amygdalin at 890 cm^−1^, in the case of films with active substance, is shifted and overlapped with that of the biopolymers present in them. Another particularity observed is the decrease in the signal for the nitrile group compared to the case of the synthesized films, which for pure amygdalin (Figure 3) was much more intense, being visible in the case of all films with active substance (A1–A4, B1–B4), the clear exception being of course the control films A0 and B0. Furthermore, for these, the appearance of a signal at 450 cm^−1^ can be observed, which, as mentioned earlier, indicates the monosubstituted nature of the benzene ring in amygdalin. These, in turn, confirm the successful incorporation of amygdalin into the synthesized films.

### 2.3. TG/DTG Analysis

- *Base Components of Films*

**Figure 6:**
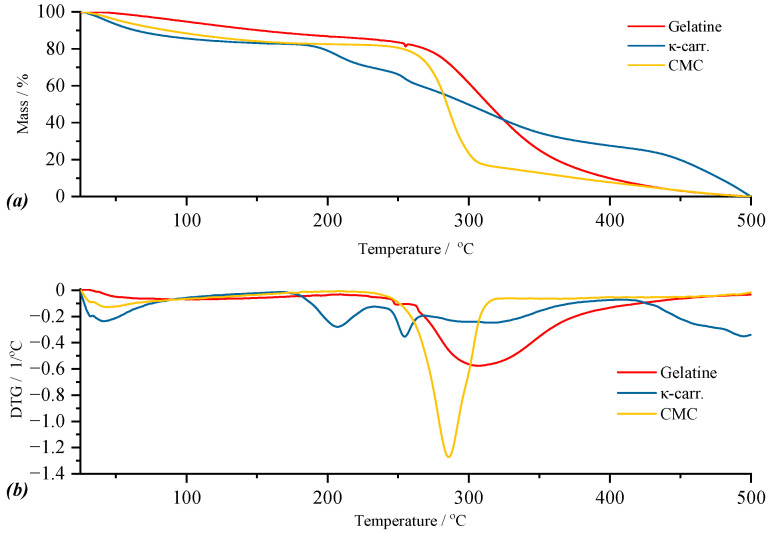
Graphical representation of TG (**a**) and DTG (**b**) curves for κ-carrageenan, gelatin, and carboxymethyl cellulose.

For a good understanding of the thermal behavior of the synthesized films, but also to facilitate the interpretation of their results, first, the thermal analysis of the base components will be discussed, whose TG and DTG curves are presented in Figure 6 (a and b, respectively). Thus, for κ-carrageenan, the TG curve indicates four thermal processes, for gelatin, three thermal processes, while for CMC, two thermal processes can be observed (Figure 6a). According to the DTG curve of κ-carrageenan, the first thermal process is included in the temperature range of 36.65–96.79 °C, in which the mass loss of 9.46% can be attributed to the elimination of water present as humidity. The mass loss of 6.10%, corresponding to the second thermal process with *T_peak_* at 193.25 °C, corresponds to the loss of polymer-bound water. These two thermal processes reconfirm the hygroscopic nature of this biopolymer, also indicated by the data communicated within the FTIR analysis. The third thermal process corresponds to the breaking of the C–SO_3_ bonds on the biopolymer skeleton [39] with a maximum of the process located at a temperature of 249.51 °C. The last thermal process is attributed to the fragmentation of the biopolymer skeleton that continues up to 500 °C. In the case of gelatin, the DTG curve highlights three thermal processes, following which the total mass loss is 56.01% of the mass of the sample subjected to analysis. Thus, the smallest mass loss in the case of gelatin, of 0.13%, is present in the first thermal process and, as in the case of κ-carrageenan, can be attributed to moisture loss. The second thermal process, which seems to be a side shoulder of the third process, is included in the temperature range of 255.66–258.35 °C, in which the mass loss is 0.69%. The last thermal process starts simultaneously with the completion of the second process. For the latter, the mass loss is 54.59% of the mass of the sample under analysis for a *T_peak_* of the process located at 359.08 °C. These two processes, located in the temperature range of 255.66–439.67 °C, can be attributed to hydrolysis and oxidation reactions [40,41] as a result of the decomposition of constituent amino acids such as glycine and proline [42]. For the CMC sample, the DTG curve highlights two thermal processes as follows: the first located in the temperature range of 41.11–69.70 °C represents water as humidity, this process being associated with a mass loss of 3.68% of the mass of the sample under analysis; the second thermal process is due to the actual decomposition of carboxymethyl cellulose which, according to the literature data [43], involves the disintegration of the polymer chain by breaking the glycosidic bonds but also the loss of functional groups linked to the chain.

- *Active Substance—Amygdalin*

**Figure 7:**
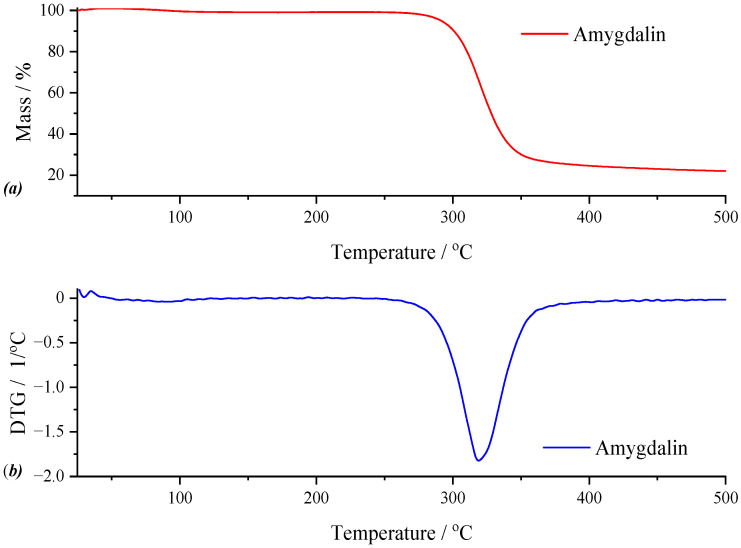
TG (**a**) and DTG (**b**) curves of amygdalin.

For the active substance, the results of the thermogravimetric analysis are graphically represented in Figure 7. Both the TG curve and the DTG curve highlight two thermal processes. Thus, the first thermal process, although with low intensity in the temperature range of 42.09–129.23 °C, results in a mass loss of 1.47%, which represents the loss of moisture. The second thermal process, practically the most intense observable on the DTG curve, can only be the decomposition of amygdalin, with a mass loss of 73.71% for the maximum of the process, located at 347.35 °C. The remaining residue, accounting for 26.29% of the initial sample mass, suggests the presence of thermally stable components. These may result from incomplete decomposition of the active compound or carbonaceous residue formation, both of which can impact the material’s degradation behavior.

- *Type A Films*

**Figure 8:**
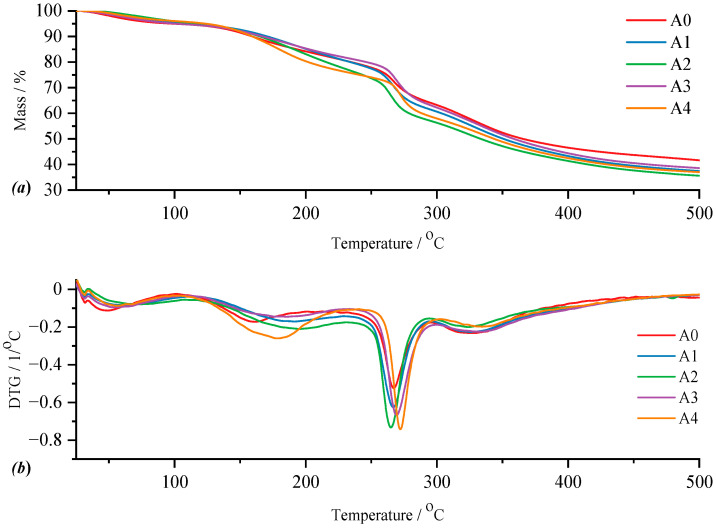
Comparative representation of TG (**a**) and DTG (**b**) curves for the control film (A0) and films with active substance (A1–A4).

For type A films, both for the control film and the films with active substance, the TG and DTG curves are represented in Figure 8. Thus, for film A0, the control film, the DTG curve highlights four thermal processes, following which the total mass loss is 54.31% of the mass of the sample subjected to analysis. The four thermal processes can be attributed as follows: the first to the loss of moisture in the temperature range of 40.46–101.14 °C observing a mass loss of 4.73%; the second as a result of the elimination of water trapped in the polymer matrix of the film and for which the mass loss is 12.46% for a *T_peak_* of the process located at 158.83 °C; the breaking of the C–S interatomic bond [44] characterized by the elimination of the sulfonic group attached to the polymer chain results in a mass loss of 17.68% in the third thermal process within the temperature range of 222.70–298.43 °C; the fourth thermal process being attributed to the actual decomposition of the film through the continuous decomposition of the carrageenan matrix but also of the rest of the components resulting in a mass loss of 19.44% for a *T_peak_* of the process at 365.54 °C. In the case of film A1, the elimination of water as humidity takes place within the first thermal process with a mass loss of 4.91% for *T_onset_* at 40.23 °C, while the elimination of “bound” water takes place within the second thermal process, for which *T_onset_* is 118.15 °C, the mass loss being 14.56. The disintegration of the carrageenan polymer chain occurs in the third thermal process with *T_peak_* at 268.70 °C for a mass loss of 18.43%. This decomposition continues in the fourth thermal process, where the final decomposition of the analyzed film also occurs, for which the mass loss is 18.97 °C with the temperature limits of 299.81–415.87 °C. For sample A2, the *T_onset_* of the first thermal process is at 40.41 °C, and the mass loss associated with this process is 4.71%, while the *T_onset_* of the second thermal process is located at 119.06 °C, for a loss of 17.47% of the mass of the sample under analysis. In the case of the third thermal process, the mass loss of 16.52% is associated with the *T_peak_* of the process located at 266.73 °C. The last thermal process in which the mass loss is 17.14% presents a *T_onset_* of 290.91 °C and a *T_endset_* of 408.40 °C. Film A3 is characterized by a *T_onset_* of 41.02 °C and a mass loss of 4.57% within the first thermal process, while the second thermal process presents a *T_onset_* of 112.60 °C and a mass loss of 18.04%. For the thermal process attributed to the elimination of sulfonic groups, the third process observable on the DTG curve in Figure 8b, *T_peak_* is located at 271.08 °C, presenting a mass loss of 16.40% of the mass of the sample subjected to analysis. The last thermal process in the case of film B3 presents a mass loss of 21.93%, being within the temperature range of 292.03–425.68 °C. Finally, the four thermal processes can also be observed in the case of film A4 as follows: the mass loss in the first thermal process is 4.02% and *T_onset_* is located at 40.85 °C while the mass loss of the second process is 20.65% for *T_onset_* of 107.00 °C and *T_endset_* 240.89 °C; the breaking of C–S bonds is followed by a mass loss of 16.57% at a *T_peak_* of 274.06 °C while the mass loss following the last thermal process which presents a *T_onset_* of 304.17 °C and a *T_endset_* 431.82 °C is 17.58%.

**Figure 9:**
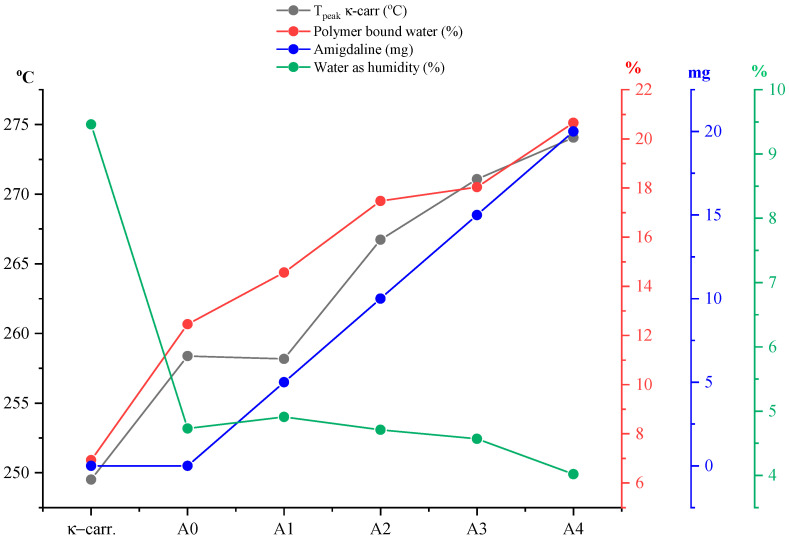
Graphical representation of the percentage of water loss as humidity, but also water bound to the polymer matrix, as well as the *T_peak_* values of the process corresponding to the disintegration of κ-carrageenan (C–S bond breakage) in type A films in relation to the amount of incorporated active substance and pure κ-carrageenan. These results demonstrate that increasing amygdalin concentration elevates the decomposition temperature, suggesting increased thermal stability.

Analyzing more carefully the appearance of the signals of the thermal processes on the DTG curve (Figure 8b), a variation can be observed in terms of *T_onset_* as well as *T_peak_* of the given process. Representing (Figure 9) the percentage of water loss graphically as humidity but also water bound to the polymer matrix, as well as the *T_peak_* values of the process corresponding to the disintegration of κ-carrageenan in relation to the amount of incorporated active substance and pure κ-carrageenan, a variation in the thermal behavior of the analyzed films can be observed much better. Knowing that type A films are obtained from a 1:1 ratio of κ-carrageenan and gelatin, it can be seen that the A0 film, more precisely its composition, has a direct impact on the positioning of the *T_peak_* of κ-carrageenan. Thus, in the case of this film, a slight shift of the process peak towards a higher temperature can be seen, as a result of the presence of gelatin. As is well known, an impurity can influence the melting point of a pure substance, either by decreasing or increasing its value. Therefore, it can be said that gelatin, even if in a 1:1 ratio, together with that drop of glycerol, acts as an “impurity” for κ-carrageenan, thus contributing to the shift of its *T_peak_* towards a higher temperature. Moreover, including the addition of the active substance, amygdalin, has a direct impact on the shift of *T_peak_* from κ-carrageenan towards a higher temperature, this trend being observed especially as the amount of amygdalin increases, A0 < A1 < A2 < A3 < A4. These statements can be easily confirmed by the appearance of the curve for *T_peak_* from carrageenan presented in Figure 9. Thus, comparing only the case of sample A0 and A1, it can be deduced that practically the greatest impact on the thermal behavior of the analyzed film is amygdalin, this component being the only one that varies. Therefore, the increase in the amount of incorporated amygdalin primarily increases the amount of water that the polymer matrix can “bind,” but at the same time causes a decrease in the amount of water present as moisture. Knowing that amygdalin has a saccharide portion in its structure, this fragment may increase the formation of H bonds with free water, thus ensuring its better retention as the film dries.

- *Type B Films*

**Figure 10:**
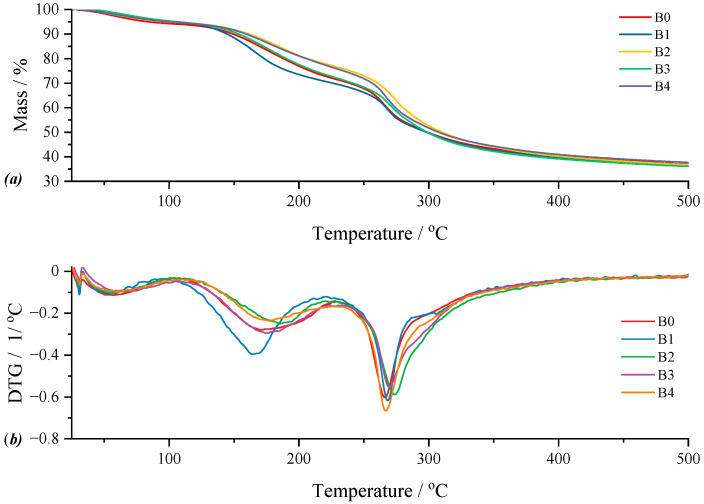
Comparative representation of TG (**a**) and DTG (**b**) curves for the control film (B0) and films with active substance (B1–B4).

The graphical representation of the thermogravimetric analysis data for type B films, including the control film, can be seen in Figure 10. For the control film (B0), the curve highlights three thermal processes resulting in a total mass loss of 58.65% of the mass of the analyzed sample. Of these, the first thermal process can be attributed to moisture loss, resulting in a mass loss of 4.49% in the temperature range of 40.86–90.86 °C. This, in turn, is followed by the loss of water trapped by the polymer matrix at a *T_peak_* of 170.15 °C of the process with a mass loss of about 22.45%. The last observable process on the DTG curve (Figure 10b) is attributed to the decomposition of the polymer matrix of the film, a stage that begins with the breaking of the C–S intermolecular bond in κ-carrageenan and continues with the thermal decomposition of its constituents. Following this last thermal process, the resulting mass loss is 31.71% in the temperature range of 233.27–415.54 °C. For film B1, according to the corresponding DTG curve, three thermal processes can be observed. Thus, the thermal effect associated with the loss of water as humidity is attributed to the mass loss of 4.15% for a *T_peak_* of the process located at 56.69 °C. The loss of water captured by the film is 24.05% in the second thermal process, whose *T_onset_* is 122.92 °C and *T_endset_* is 223.62 °C, respectively, while the *T_peak_* associated with the process is 165.02 °C. The decomposition of the polymer film begins in the third thermal process at 229.09 °C, which, as can be seen from Figure 10b, presents a maximum at 270.16 °C, reaching a *T_endset_* of 403.73 °C. Thus, it can be said that in this process, the decomposition of the active substance also takes place, and a delimitation cannot be made that would accurately specify the beginning of the amygdalin decomposition in the analyzed film. This fact can also be confirmed by the percentage of mass loss, 31.74%, which is higher compared to that of the control film and which indicates the inclusion of the decomposition of the active substance within the appropriate limits (mentioned above). In the case of film B2, we can find again the same three processes for which the following data can be communicated; the mass loss of 4.95% for a *T_peak_* of 60.29 °C can be attributed to the loss of water as humidity while the loss of 16.95%, for a *T_peak_* of 184.97 °C, to the water “captured” by the matrix, within the first and second thermal processes, respectively. The last thermal process corresponds to the decomposition of both the polymer matrix and the active substance, resulting in a mass loss of 37.43% of the mass of the sample subjected to analysis, for the temperature range of 229.42–406.88 °C. For film B3, the loss of water as moisture is followed by a mass loss of 5.25%, which occurs within the first thermal process for which *T_peak_* is positioned at 60.55 °C. In contrast, the water “bound” to the matrix produces a mass loss of 21.93%, this thermal effect being located within the second thermal process for which *T_onset_* is 130.27 °C and *T_endset_* is 225.46 °C. The last thermal process, within the temperature range of 234.34–406.59 °C, corresponds to the proper decomposition of the film and amygdalin, respectively, thus resulting in a mass loss of 32.98%. Finally, for sample B4, the first thermal process is given by the loss of water as moisture within the temperature range of 41.24–96.97 °C, the mass loss being 5.24% of the mass of the sample subjected to analysis. The second process, which corresponds to the elimination of water trapped in the polymer matrix, for which *T_peak_* is located at 173.55 °C, results in a mass loss of 17.02%. The last thermal process observable on the DTG curve initiates the disintegration of the film matrix and continues with the decomposition of the active substance, this being a continuous process without any clear delimitation between these two steps. For this, the mass loss is 35.07%, the lower limit is 230.87 °C, and the upper limit is 399.62 °C.

**Figure 11:**
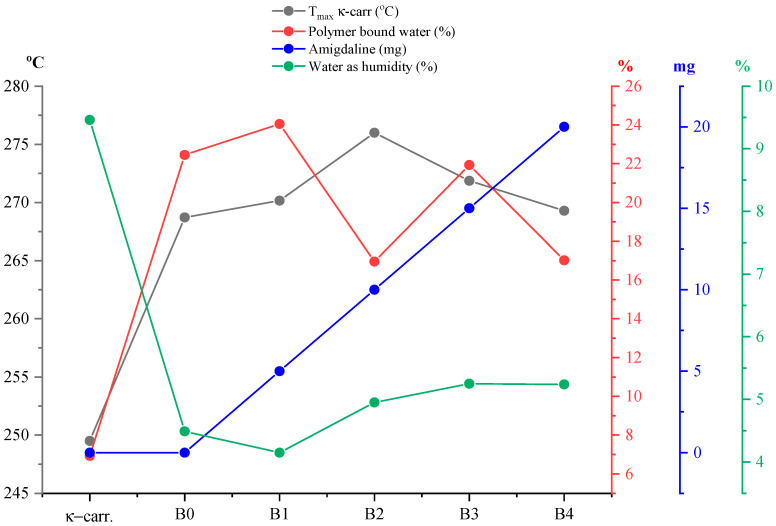
Graphical representation of the percentage of water loss as humidity, but also water bound to the polymer matrix, as well as the *T_peak_* values of the process corresponding to the disintegration of κ-carrageenan (C–S bond breakage) in type B films in relation to the amount of incorporated active substance and pure κ-carrageenan. These results demonstrate that increasing amygdalin concentration elevates the decomposition temperature, suggesting increased thermal stability.

To better detail the thermal behavior of type B films, but also to determine the impact that the variation in the secondary biopolymer may have, the same analysis was approached as in the case of type A films. Thus, in Figure 11, the percentage of water loss was represented both as humidity and water related to the polymer matrix, but also the *T_peak_* values of the κ-carrageenan disintegration process in relation to the amount of incorporated active substance and pure κ-carrageenan. Thus, regarding Figure 11, a rather interesting variation in the represented parameters can be observed. Knowing that the films are formed from the same mass ratio of the two biopolymers and that the only variable parameter was the amount of active substance chosen to be incorporated into the film, at first, we can say that this varied behavior would be due to the active principle. By comparing with the behavior of type A films in Figure 9, it can be observed that in the case of type B films, the curve of the percentage of water as humidity has a slightly ascending evolution. Therefore, it can be said that the replacement of the secondary bipolymer, in this case CMC, influences the water, as humidity, which the film loses due to this cellulose derivative, which, as is well known, is used in industry as a water retention agent. Thus, even if the physical appearance suggests that the films analyzed were dried, at the molecular/microscopic level, the thermogravimetric analysis indicates that the type B films still present a greater amount of water than the type A ones. It can be said that CMC in this case acts as a water retention agent due to the multitude of hydroxyl groups that can form hydrogen bonds with water molecules, thus preventing drying beyond the point of disintegration of the films, which explains the increased elasticity observed in this film type. Furthermore, regarding the curve for polymer bound water, it can be observed that, while it has an ascending character in type A, in the case of type B it shows a slight increase in the case of sample B1, followed by a drastic decrease in the case of sample B2, a behavior that follows the trend: B0 < B1 > B2 < B3 > B4 ≈ B0. A possible explanation could be that the composition of the samples has a direct impact on this parameter, and it can be said that the amount of active substance determines the observed variation, knowing that this is the only component varied in the case of type B films. Although the films do not show a well-defined thermal process that can be attributed with certainty to the disintegration of κ-carrageenan (breaking the C–S bond), the representation of *T_peak_* for pure carrageenan was chosen compared to *T_peak_* of the thermal process that encompasses its decomposition and the active substance. Thus, as can be observed, a shift in the *T_peak_* value can be observed depending on the amount of amygdalin, a behavior also observed in the case of type A films.

Both types of films exhibit a multi-step thermal decomposition, differing only in the number of thermal processes observable on the corresponding DTG curves. As for type A films, they consistently exhibit four thermal processes: water loss in the form of humidity, elimination of bound water, C–S bond cleavage, and degradation of amygdalin and the polymer matrix. In contrast, type B films exhibit only three thermal processes, the most pronounced thermal process including both the C–S bond cleavage of the base polymer and the actual decomposition of both amygdalin and the polymer matrix. Thus, this difference indicates a greater degree of complexity in the thermal behavior of type A films, a possible cause being the peptide nature of gelatin itself. In contrast, type B films show a higher loss of bound water (up to ~25%) compared to type A films (~21%), indicating that CMC has a higher water retention capacity, due to the hydroxyl and carboxyl groups. Therefore, it can be said that a stronger water–polymer interaction occurs in the case of type B films, giving them a more pronounced flexibility compared to type A films, as previously mentioned in Section 2.1. Regarding the active substance, thermogravimetric data indicate that amygdalin has an impact on the mass loss, which is more pronounced in the case of type B films, possibly due to the lower number of interactions between CMC and amygdalin, which, in turn, can somewhat stabilize or even thermally protect the compound.

### 2.4. In Vitro Testing for Bactericidal Activity of Amygdalin-Doped Films

The determination of the bactericidal activity of the films with active substance, both type A and type B, was carried out in two series, simultaneously carrying out a control sample, consisting only of the culture medium and the bacterial inoculum (as can be seen in Table 2). Thus, according to those mentioned earlier, each sample of amygdalin-doped polymeric film (including a control sample for each type) was immersed in bacterial suspension and incubated at 37 °C under agitation for 24 h. The viability of the bacterial cells was determined using the TTC assay, and the viability of the cells was measured spectrophotometrically at 485 nm. When assessing cell viability via the TTC assay, the intensity of the produced formazan color correlates directly with the viability of the bacterial cells. This viability was influenced by the specific polymeric films tested and their respective antibacterial effects.

**Figure 12:**
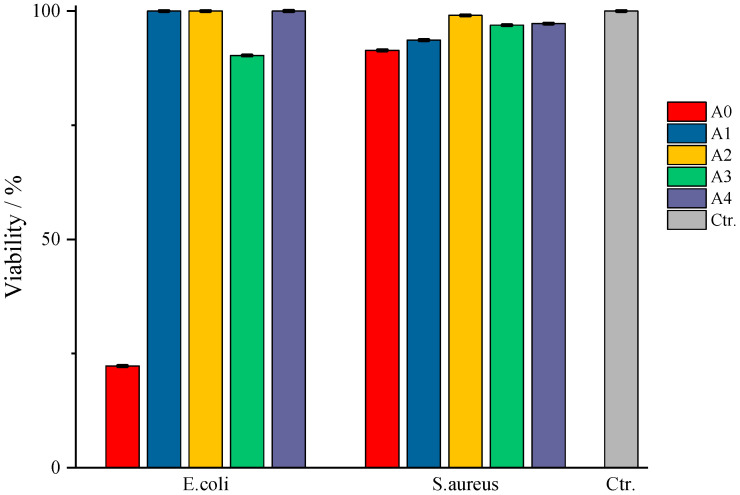
Schematic representation of the results of in vitro tests for type A films.

According to Figure 12, in which the values obtained from the TTC viability assay are represented compared to the control sample, type A films do not show bactericidal activity against the tested strains. An exception to this observation is the case of sample A0, in which the bacteria show a viability of 20%, respectively, an efficiency of the A0 films of 80% against *E. coli*, and sample A3, where an 80% viability of the same pathogen is observed. Even though sample A0 does not present entrapped amygdalin, it still manages to prevent the multiplication of the pathogen quite well. A possible explanation could come from the composition of the film itself, more precisely, due to κ-carrageenan. In this case, this biopolymer exerts its antibacterial property against *E. coli*, data from the literature indicates inhibiting activity against *Staphylococcus aureus*, *Escherichia coli*, *Staphylococcus aureus*, *Penicillium citrinum*, etc., depending on its concentration [45,46]. In the case of films with amygdalin, tested against the same pathogen, the difference of which lies in the amount of amygdalin entrapped but on the same biopolymer base, the pathogen is practically not affected by the presence of the active substance nor by the biopolymer matrix. Although some studies in the literature indicate that crude extracts of amygdalin, often containing alkaloids, flavonoids, etc., from various seeds, show a slight bactericidal activity against some pathogens, including *E. coli* [47]; in the present study, pure entrapped amygdalin did not exhibit bactericidal activity against *E. coli*. A likely explanation is the ability of the bacteria to express β-glucosidase activity, specifically the periplasmic enzyme BglX [48], which hydrolyzes glycosidic bonds. This enzyme efficiently cleaves the β-glycosidic bond between the glucose units and the aglycone moiety, converting amygdalin to glucose and other metabolites. As a result, instead of acting as an antimicrobial agent, amygdalin is enzymatically degraded, thus serving as a carbon source for *E. coli*, which can subsequently attack the base biopolymer. Moreover, this periplasmic protein has a preference for galactose units, present in κ-carrageenan, as reported in a study by Ngo, Lorna et al. [48]. Therefore, once β-glucosidase begins to hydrolyze the glycosidic bonds in carrageenan, the structural integrity of the film is compromised, thereby diminishing and nullifying its antibacterial function. Regarding antibacterial activity against *S. aureus*, its absence may again be due to the enzyme, this time involving phospho-β-glucosidases, which again can act on glycosidic bonds [49], both those in amygdalin and those in κ-carrageenan, as described in the lines above.

**Figure 13:**
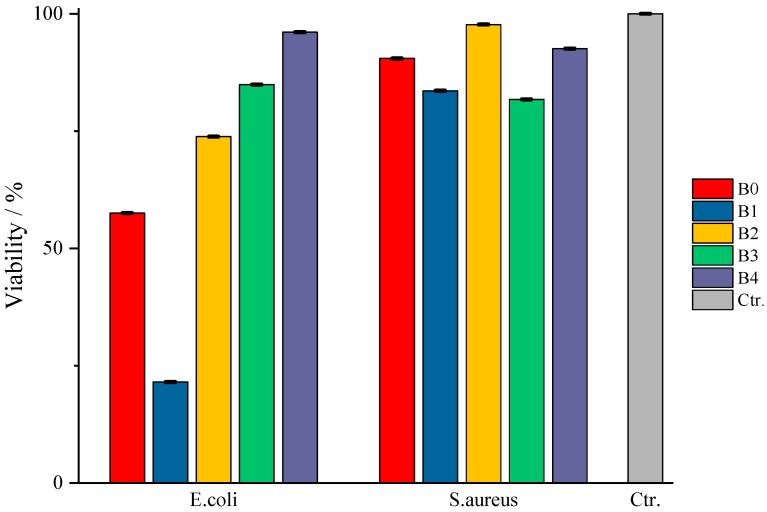
Schematic representation of the results of in vitro tests for type B films.

Regarding type B films, the results obtained are presented in Figure 13. For the samples tested on *E. coli* culture, a decrease in the evolution of the active substance’s action can be seen depending on its quantity in the corresponding sample. Thus, in the case of sample B0, even though it does not have an incorporated active substance, it still exhibits a bactericidal action, as was the case with sample A0, but with a much higher cell viability of ≈60%, i.e., an efficiency of 40% of the polymeric material. This drastic difference in the behavior of the control samples can only be the result of their composition, there being the possibility that the secondary biopolymer is the answer to this phenomenon. Gelatin, the secondary biopolymer of type A, cannot be “digested/attacked” by *E. coli*, since they do not produce gelatinase [50]; this pathogen becomes available for “attack” during incubation by the bactericidal activity of κ-carrageenan. In contrast, the CMC in type B films could become susceptible to attack by the periplasmic protein of this pathogen due to the presence of glycosidic bonds and therefore could become a “source of nutrients”, thus offering the possibility for this pathogen to attack, to some extent, the basic bipolymer, carrageenan, resulting in a notable difference between the bactericidal efficacy of samples A0 and B0. Moreover, as the amount of active substance increases, the bactericidal efficacy of the films decreases in the order B1 < B2 < B3 < B4 because amygdalin, prone to glycosidic bond breakage, together with the secondary biopolymer, as proposed earlier, prevents the exercise of the bactericidal activity of carrageenan. Comparing the viability values, it can be observed that sample B1 presents a much more pronounced bactericidal efficacy compared to the control sample. There is a possibility that amygdalin, due to its structure, is much more easily attacked by *E. coli*, and its small amount in sample B1 does not become the “sufficient amount” of nutrient that would ensure the survival of the pathogen when “attacked” by the basic polymer. Thus, as the amount of amygdalin increases, the pathogen also has a larger amount of “amygdalin-nutrient” available, thus facilitating the attack on both biopolymers of the film, resulting in the observed behavior of the tested samples. In the case of the test for *S. aureus*, the same behavior can be observed as in the case of type A samples, the absence of efficacy on this bacterial culture being again linked to the secretion of phospho-β-glucosidase.

## 3. Conclusions

Considering the usefulness of biopolymers in the medical field, including wound dressings, the composite films synthesized in this study can become a starting point in what represents the development of new types of bio-dressings. Knowing that the biopolymer matrix of the films has a direct impact on their physical appearance, those with CMC (type B) presenting a sticky character make this property an advantage regarding their possible use as a dressing for skin wounds. Moreover, their short drying time offers them another advantage, this parameter being desirable and crucial for a larger-scale production compared to that at the laboratory level. The results of the FTIR analysis manage to highlight both the successful incorporation of amygdalin and the absence of interactions between this active principle and the components of the polymer matrix of the synthesized films. In the thermogravimetric study, the influence of the secondary biopolymer on the amount of water that the synthesized film can bind was evidenced, as well as the influence on the shift of the *T_peak_* value of the thermal process associated with the decomposition of the polymer base. The amount of amygdalin incorporated also contributed to the observed effect. Also, the results of this study indicate a thermal stability in the range of 25–100 °C, both for the active substance and the biopolymer film. In contrast, the results of the in vitro study indicate an inactivity of the type A films doped with amygdalin against *E. coli* and *S. aureus*, the exception being sample A0 (without active substance against *E. coli*). Sample A0 becomes a witness to the antibacterial properties of the base polymer, κ-carrageenan, mentioned in the specialized literature. As for type B, it can be said that they do not show bactericidal activity against *S. aureus* but rather against *E. coli*. In the case of *E. coli*, it was highlighted that bactericidal activity decreases as the amount of amygdalin increases. The case of sample B1, with an efficiency of 80%, allows us to say that the small amount of incorporated amygdalin (5 mg) per synthesized film could be exploited in the field of wound dressings in the case of *E. coli* infections. In conclusion, it can be said that type B films (κ-carrageenan-CMC based) show promising potential in the development of wound dressings, due to the positive bactericidal response of amygdalin on *E. coli*, as well as their hydration capacity, which can provide greater hydrophilicity and flexibility and better initial bioactivity in hydrated environments. These results urge us to conduct more in-depth research in terms of testing them on other bacterial cultures, common to wounds, as well as experimenting with other secondary polymers, to develop a material intended to be used as a bio-wound dressing.

## 4. Materials and Methods

### 4.1. Chemicals

The polymer matrix of the biopolymer films was obtained using κ-carrageenan (M.W. of 788.647 g/mol) by Acros Organics (Geel, Belgium), sodium salt of carboxymethyl cellulose by Sigma-Aldrich (Merck KGaA, Darmstadt, Germany), CAS No.: 9004-32-4, gelatin by Sigma-Aldrich (Merck KGaA, Darmstadt, Germany), CAS Number 9000-70-8 and as a plasticizer, glycerol (M.W. 92.10 g/mol) by CHIMREACTIV (Ion Creanga, Romania). The active substance chosen to be incorporated into the biopolymer films was amygdalin, from apricot kernels, produced by Sigma-Aldrich (Merck KGaA, Darmstadt, Germany), CAS.No.: 29883-15-6.

### 4.2. Methods

- *Synthesis of biopolymer films*

The biopolymer films were obtained using the methodologies as described in our previous publications on biopolymer-based drug delivery systems [51,52,53,54]. The films are composed of a base biopolymer (κ-carrageenan), a secondary one, and a plasticizer (glycerol), thereby introducing a novel approach by using either gelatin or carboxymethyl cellulose (CMC) as the secondary biopolymer in the polymer matrix. Following the conducted experiments, a series of combination ratios and a variety of films were obtained. Among these, two types of films were chosen as carriers for amygdalin, being selected as potential candidates for wound dressing material.

The first type of film (type A films) was obtained by dissolving κ-carrageenan and gelatin (in a mass ratio of 1:1, *w*/*w*) in 10 mL of distilled water under stirring at 500 rpm until a homogeneous solution was obtained, to which a drop of glycerol was subsequently added. It should be noted that the dissolution of the biopolymers was carried out in stages that involve dissolving the carrageenan in water at 85 °C, bringing the obtained solution to a temperature of 60 °C, adding the calculated amount of gelatin, and finally adding glycerol under continuous stirring for 5 min. This process represents the obtaining of the biopolymer matrix base of type A films.

For the second type of films (type B), the process for obtaining the biopolymer matrix base is similar to that described for type A, the combination ratio between the base biopolymer (κ-carrageenan) and the secondary biopolymer (CMC) being 1:1 *w*/*w*. Thus, the calculated mass of carrageenan was first solubilized at 85 °C and under magnetic stirring (500 rpm), after which the solution obtained was cooled again, but this time to 50 °C, followed by the addition of CMC, and finally, one drop of glycerol was added.

- *Amygdalin-doped films*

To obtain the amygdalin-doped films, both type A and type B, the corresponding stock solutions were used. Thus, four samples of stock solution were prepared, for both types of biopolymer base, to which amygdalin was added so that the resulting films incorporated 5 mg, 10 mg, 15 mg, and 20 mg, respectively, per film obtained. Knowing that 10 mL of distilled water was used in the preparation of the polymer base and that the quantities of amygdalin mentioned above were used, it can be said that the resulting film samples have a concentration of 0.5 mg (for A1 and B1), 1.0 mg (for A2 and B2), 1.5 mg (for A3 and A4), and 2.0 mg (A4 and B4) of active substance per mL of stock solution. The homogeneous solutions were poured into a glass Petri dish with a diameter of 5 cm and dried in an oven at 45 °C.

- *FTIR analysis*

The polymeric films were cut into small sections and analyzed directly without further chemical treatment, and placed in direct contact with the ATR crystal. Analysis was carried out on a Shimadzu FT-IR IRTracer-100 spectrometer with ATR performed in the range 4000–400 cm^−1^, data collection being performed after 20 recordings at a resolution of 4 cm^−1^.

- *TG/DTG analysis*

Sample analysis was performed on a METTLER TOLEDO thermogravimetric analyzer, model TGA/DSC3+, over a temperature range of 25–500 °C with a heating rate of 10 °C∙min^−1^ in an air atmosphere (with a flow rate of 50 mL/min) in open aluminum crucibles. Prior to analysis, the polymeric film samples were cut into small, uniform pieces to ensure consistent thermal contact. Approximately 5–10 mg of each sample was accurately weighed and placed in an open alumina crucible.

- *Microorganisms and growth conditions*

Two bacterial strains, Gram-positive *Staphylococcus aureus* ATCC 29213 and Gram-negative *Escherichia coli* ATCC 25922, were used to determine the antibacterial effect of polymeric films. Bacterial glycerol stocks were thawed on ice and grown overnight on Tryptic Soy Agar (agar 15 g/L, casein peptone (pancreatic) 15 g/L, sodium chloride 5 g/L, soya peptone (papainic) 5 g/L). One isolated colony from each strain was transferred to Tryptic Soy Broth (casein peptone (pancreatic) 17 g/L, dipotassium hydrogen phosphate 2.5 g/L, glucose 2.5 g/L, sodium chloride 5 g/L, soya peptone (papain digest) 3 g/L) and incubated overnight. Both stains were grown aerobically at 37 °C and 250 rpm (for liquid culture) in a thermostatic orbital shaking incubator (Heidolph Unimax 1010, incubator). 2,3,5-triphenyltetrazolium chloride solution, used for the viability assay, was prepared at a concentration of 5 mg/mL and sterilized using 0.22 µm syringe filters and kept at 4 °C before use. Both dehydrated growth media and 2,3,5-triphenyltetrazolium chloride were purchased from Merck.

- In vitro *study. Antibacterial effect of amygdalin-doped films*

The antibacterial assessment was performed based on the method of Galiano et al. [55] with some modifications as follows: bacterial suspensions (5 mL) adjusted to an optical density at 600 nm (OD_600_) of 0.1 in Tryptic Soy were pipetted onto a six-well plate, and each type of polymeric film was immersed in that bacterial suspension and incubated at 37 °C under agitation for 24 h. The same working protocol was used for control wells containing only the bacterial suspensions. The viability of the bacterial cells was determined using the TTC (2,3,5,-triphenyltetrazolium chloride) assay as described by Moussa et al. [56]. In the presence of bacteria, TTC is converted into red formazan, a process that correlates directly with the number of viable, active cells. As a result, the TTC test is regarded as a relatively rapid method for assessing the antibacterial efficacy of antimicrobial agents. The TTC assay typically yields susceptibility results in under 12 h and requires a lower bacterial count for accurate evaluation. TTC is a colorless or pale yellow tetrazolium salt that acts as an artificial electron acceptor. In living (metabolically active) cells, dehydrogenase enzymes present in the bacterial cytoplasm reduce TTC into formazan, a red, insoluble compound. The intensity of the red color (quantified spectrophotometrically at 485 nm using a multimode microplate reader, Biotek Synergy H1) is directly proportional to the number of viable cells.

Therefore, after 24 h of incubation, the polymeric films were taken out of the wells, and 100 µL of bacterial suspensions from each sample and control were transferred into a sterile 96-well plate. In total, 50 µL of TTC 5 mg/mL was added, and the plate was incubated, under agitation, for one hour in the dark. Afterwards, the microplate was centrifuged at 4000 rpm for 5 min (Hettich Universal 320 R), the supernatant was discharged, and the formazan was suspended in 50% ethanol and solubilized, followed by another centrifugation step (4000 rpm for 5 min). The supernatant was transferred into an empty sterile 96-well plate, and the viability of the cells was measured spectrophotometrically at 485 nm. The viability was calculated based on the control.

## Figures and Tables

**Table 1:** Physical appearance of the obtained films.

Type A Films	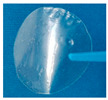 A0	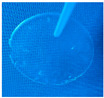 A1	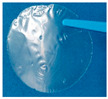 A2	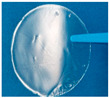 A3	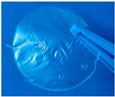 A4
Type B Films	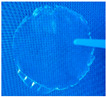 B0	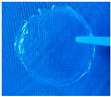 B1	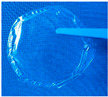 B2	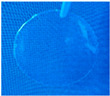 B3	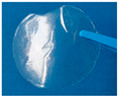 B4

**Table 2:** Determination of the bactericidal activity of type A and type B films. Physical appearance of the samples at T0 (the time of inoculation of the samples with pathogens) and T24 (after 24 h of incubation and exposure to pathogens); CONTROL—culture medium and the bacterial inoculum.

	*E. coli*		*S. aureus*	
	Type A Films	Type B Films	Type A Films	Type B Films
**T0**	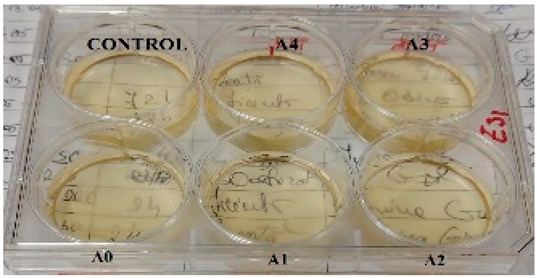	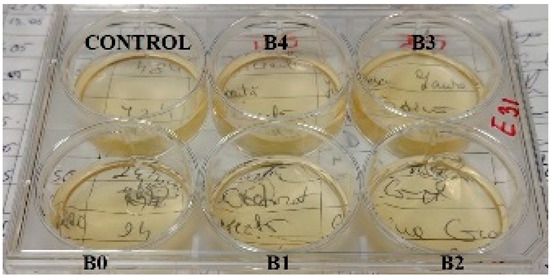	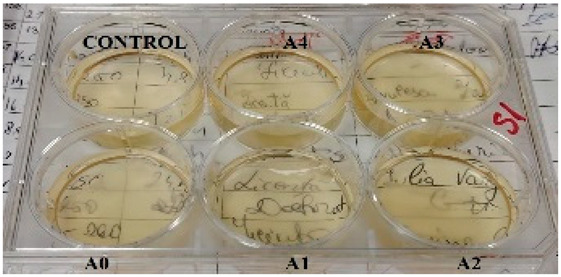	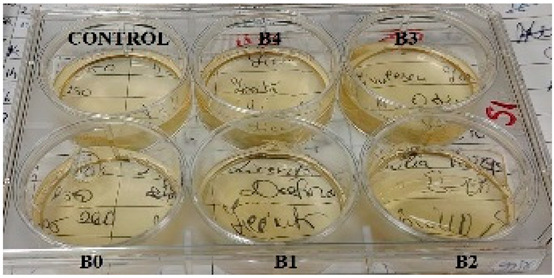
**T24**	*** 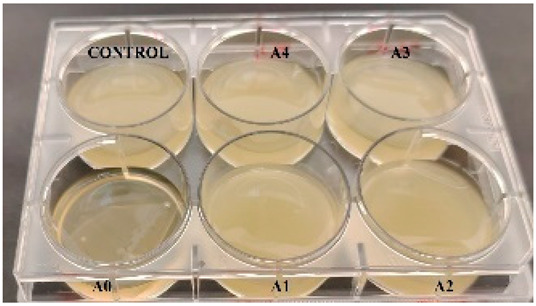 ***	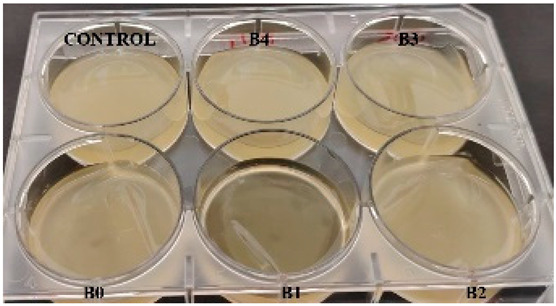	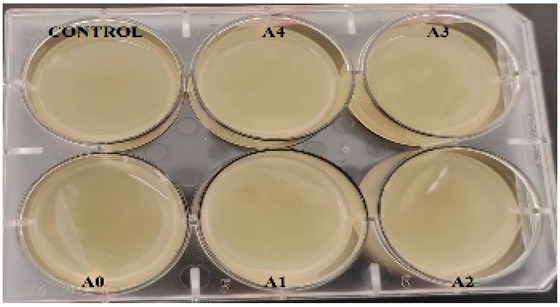	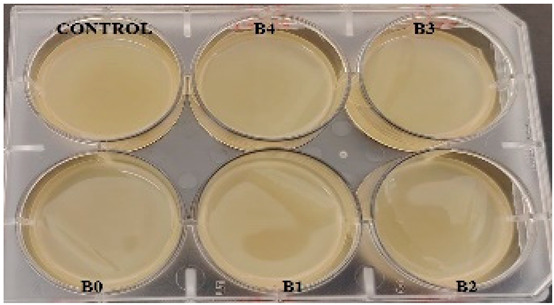

## Data Availability

The original contributions presented in this study are included in the article. Further inquiries can be directed to the corresponding author.
